# Genomic Characterization of Sulphite Reducing Bacteria Isolated From the Dairy Production Chain

**DOI:** 10.3389/fmicb.2018.01507

**Published:** 2018-07-05

**Authors:** Conor J. Doyle, Paul W. O'Toole, Paul D. Cotter

**Affiliations:** ^1^Teagasc Food Research Centre, Cork, Ireland; ^2^School of Microbiology, University College Cork, Cork, Ireland; ^3^APC Microbiome Ireland, Cork, Ireland

**Keywords:** sulphite reducing bacteria, SRCs, sulphite reducing clostridia, genomics, food microbes

## Abstract

Anaerobic sporeformers, specifically spoilage and pathogenic members of the genus *Clostridium*, are a concern for producers of dairy products, and of powdered dairy products in particular. As an alternative to testing for individual species, the traditional, and still current, approach to detecting these sporeformers, including non-spoilage/non-pathogenic species, in dairy products has involved testing for a sulphite reducing phenotype [Sulphite reducing Clostridia (SRCs)] under anaerobic conditions. This phenotype is conserved throughout the Order Clostridia. Unfortunately, however, this phenotype is exhibited by other sulphite reducing bacteria (SRBs) also, potentially leading to potential for false positives. Here, this risk was borne out through the identification of several SRBs from industry samples that were identified as *Proteus mirabilis* and various *Bacillus/Paenibacillus* sp. Genome wide comparison of a number of representative SRCs and SRBs was employed to determine phylogenetic relationships, especially among SRCs, and to characterize the genes responsible for the sulphite reducing phenotype. This screen identified two associated operons, i.e., *asrABC* in SRCs, and *cysJI* in *Bacillus/Paenibacillus spp*. and *P. mirabilis*. This screen identified spp. belonging to *sensu stricto, Lachnospiraceae* and Cluster XIV of the Clostridia all producing the SRC phenotype. This study highlights the inaccuracy of the industry standard SRC test but highlights the potential to generate an equivalent molecular test designed to detect the genes responsible for this phenotype in clostridia.

## Introduction

Raw milk is populated by a variety of metabolically and taxonomically diverse bacteria, the majority of which are inactivated by commercial pasteurization (Wells-Bennik et al., 2016). While this process reduces the overall bacterial load and diversity of the milk, it selects for thermoduric and, in particular, sporeforming, bacteria. This is notable as sporeforming bacteria, including many anaerobic sporeformers, are present in niches throughout the dairy chain, extending from farm to factory (Wells-Bennik et al., 2016) and are a significant concern for the dairy industry (Doyle et al., 2015). The majority of strictly anaerobic sporeformers of concern to the dairy industry belong to the *Clostridium* genus, specifically to Cluster I and Cluster II, and are also known as the *Clostridium sensu stricto* (McAuley et al., 2014; Doyle et al., 2015). From a spoilage perspective, some of these *Clostridium* spp. can cause late-blowing defects in cheese due to butyric acid production (Bassi et al., 2015). *Clostridium tyrobutyricum* is most commonly associated with this defect but *Clostridium sporogenes, Clostridium butyricum, Clostridium beijerickii* and, to a lesser extent, *Clostridium tertium* may also cause or contribute to this defect include (Bermúdez et al., 2016). From a public health perspective, *Clostridium perfringens, Clostridium botulinum*, and *Clostridium tetani* are of greatest concern due to their toxigenic potential. *C*. *perfringens* is the most prevalent of these species from a foodborne illness perspective, and causes in excess of 1 million incidences of foodborne illness in the United States per annum (Scallan et al., 2011). Although, between 1998 and 2008 only one case of foodborne illness in the United States was attributed to a dairy related vector (Bennett et al., 2013), *C. perfringens* has recently been isolated throughout the dairy farm environment in Australia, including in raw milk (McAuley et al., 2014), has been detected in defective cheese in Italy (Bassi et al., 2015) and its presence in powdered infant formula (PIF) has been reported (Barash et al., 2010). In the case of *C. botulinum*, while the presence of the pathogen in PIF has been associated with two incidences of infant botulism previously, one in the United States and one in the United Kingdom in 2001, the links were not conclusively established (Barash et al., 2010). Regardless, this species remains a concern for dairy producers, particularly for those that produce products for infant consumption, as the infectious dose for botulinum spores in infant botulism is thought to be extremely low (ICMSF, 2014) and the reputational damage associated with an outbreak would likely be great. Indeed, the inaccurate reporting of the presence of *C. botulinum* in PIF originating from New Zealand has previously resulted in a significant recall (Doyle and Glass, 2013). To our knowledge *C. tetani* has not been associated with any incidences of foodborne illness associated with the consumption of dairy product, nor has it been reported to have been detected in dairy products. Nonetheless it remains of concern to producers because of its ability to produce a neurotoxin.

Because of the toxigenicity of some members of the Clostridia, coupled with the potential of some members of the *sensu stricto* to cause spoilage in dairy products, it is routine to test dairy products for the presence of these sporeformers. The test employed most frequently, primarily for historical reasons, involves the enumeration of sulphite reducing Clostridia (SRC) and relies on the ability of the majority of *Clostridium* spp. of concern to the dairy industry to reduce sulphite to sulfide (Weenk et al., 1995; Doyle et al., 2015), most frequently through use the *asrABC* operon involved in dissimilatory sulphite reduction (Czyzewski and Wang, 2012). The *asrABC* operon has previously been described in *Clostridium spp*. and *Salmonella enteria* (Huang and Barrett, 1991). However, other bacteria referred to as sulphite reducing bacteria (SRBs) may have other genes (*cysJI*) that produce the same phenotype (Standards, 2003) and result in false positives (Weenk et al., 1991; Doyle et al., 2015). Indeed, aerobic sporeformers and even Gram negative bacteria have caused such false positive results in the past (Sugiyama, 1951; Fischer et al., 2012). Ultimately, the distribution of the SRC phenotype throughout the heterogeneous *Clostridium* genus, including many species that were previously considered *Clostridium*, (Ludwig et al., 2009) is not well understood, making the relevance of the SRC assay unclear.

The objectives of this study were to determine the identity of SRCs, and SRBs, isolated from a variety of dairy sources, and to employ comparative genomics to identify genetic features common among SRCs with a view to the identification of conserved loci that could be used for alternative, DNA-based, diagnostic approaches.

## Materials and methods

### Isolation and identification of sulphite reducing isolates

Anaerobic sulphite reducing bacteria were isolated from dairy powders, cheese and raw bulk tank milk using standard protocols (Standards, 2004). This method includes a heat inactivation step (80°C for 10 min) that is intended to eliminate non-sporeforming bacteria. Black colonies were then aseptically picked and grown in pure culture in reinforced *Clostridium* media before DNA was extracted using the Mericon Bacteria plus kit (Qiagen). The 16S rRNA gene was amplified from each isolate using the CO1 and CO2 primers (Simpson et al., 2003). This PCR was conducted using the following parameters; 94°C for 5 min, followed by 30 amplification cycles, each consisting of three 1 min stages at 94°C, 60°C, and 72°C, with a final extension of 5 min at 72°C. Amplified DNA was then purified using the GenElute PCR cleanup kit (Sigma Aldrich, Wexford, Ireland) before Sanger sequencing was carried out (Source Bioscience, Waterford, Ireland). The resulting sequences were than subjected to BLAST analysis (Altschul et al., 1997) against the NCBI database with a view to determining their identity.

### Whole genome sequencing

Genomic DNA, which had been extracted as described above, was cleaned up using the Powerclean kit (Mo Bio, Carlsbad, CA). Genomic DNA was then quantified using the Qubit high sensitivity kit (Bioscience, Dublin, Ireland), prepared for sequencing using the Nextera XT library preparation kit (Illumina) and sequenced on the Illumina Miseq platform using paired-end 2 × 250 base pair reads at the Teagasc Sequencing Centre, Teagasc Food Research Centre, Moorepark. Raw reads were processed and filtered based on quality and quantity and trimmed to 200 bp with a combination of Picardtools (https://github.com/broadinstitute/picard) and SAMtools (Li et al., 2009). Quality was visualized using FastQC (Andrews, 2010). Sequences were assembled using IDBA-UD (Peng et al., 2012), removing all contigs smaller than 500 bp.

### Annotation, phylogenetic comparison and analysis of core genes of *Clostridium* genus

Assembled contigs from sequenced isolates and genome scaffolds from the NCBI genome repository were annotated using Prokka (Seemann, 2014). Global alignment of amino acid sequences was carried out using Phylophlan (Segata et al., 2013). A phylogenetic tree was created from this alignment using FastTree (Price et al., 2009). The phylogenetic tree was then visualized using Graphlan (Asnicar et al., 2015). Using the .gff files from Prokka, Roary (Page et al., 2015) was used to compare the annotated genes from all SRBs using a BLASTp threshold of 50. In addition, core genes within SRC were also identified using Roary (Page et al., 2015) setting a BLASTp threshold of 50% for both comparisons. Sequences for each species can be found on ENA under accession numbers ERS1887784, ERS1887785, ERS1887786, ERS1887787, ERS1887788, and ERS1887789.

### *In silico* screening for sulphite reducing genes among SRBs

A protein database was created containing all the annotated genomes of the SRBs listed in Table S1. For the SRC phenotype, query amino acid sequences for the *A, B*, and *C* subunits of the *asr* gene cluster from the type *C. butyricum* strain, DSM 10702, were BLASTed against this database (Altschul et al., 1997). For the non-SRC SRB blastp query searches, the amino acid sequences for the assimilatory sulphite reducing genes *cysI* and *cysJ* from *B. licheniformis* were selected as this was the most frequently isolated *Bacillus* SRB in the surveillance.

### Analysis of amino acid sequence homology in *asrABC* and *cysIJ*

The sample sequences for BLASTp hit for each gene were retrieved from the BLASTp searches and converted into fasta format and aligned using MUSCLE (Edgar, 2004) for visual inspection of conservation. Aligned sequences from each gene were visualized using Jalview (Waterhouse et al., 2009). The amino acid sequences of the A, B and C subunits of the *asr* operon were examined for the presence of conserved functional domains. Furthermore, the *cysI* and *cysJ* genes were also analyzed for conserved amino acid domains. The structure of these proteins was also modeled using Phyre2 (Kelley et al., 2015).

## Results and discussion

### Identification of SRBs in dairy products

In order to better understand the prevalence and identity of SRBs in the Irish dairy chain, 101 positive SRB isolates were identified by Sanger sequencing of the corresponding 16S rRNA gene. 77 isolates were identified as clostridia (SRCs), 19 were *Bacillus sp*., 3 isolates were *Proteus mirabilis* and 2 *Paenibacillus sp*. (Table 1). It was thus apparent that the SRBs present in the dairy chain were relatively heterogeneous, with the proportion of non-clostridia being particularly notable in light of the purpose of the assay i.e., to detect SRCs. The basis for positive phenotypes was anticipated to reflect the presence of *asrABC* operons [i.e., those associated with *Clostridium* spp. (Czyzewski and Wang, 2012)], and *cysJI* operons [i.e., those previously found in *P. mirabilis, Bacillus/Paenibacillus* and other genera (Guillouard et al., 2002; Turnbull and Surette, 2008)].

**Table 1:** SRB detected in the surveillance of raw milk and dairy products.

**ID**	**Genus/species**	**Source**	**ID**	**Genus/species**	**Source**
CD1,2 & 6	*[Clostridium] amygdalinum*	BTM	CD52, 55 & 71	*Clostridium tyrobutyricum*	Industry
CD3-5 & 7-25	*[Clostridium] bifermentans*	Industry	CD77	*Clostridium celecrecens*	Industry
CD26	*Clostridium tyrobutyricum*	BTM	CD78	*Paenibacillus*	Industry
CD27	*Clostridium algidicarnis*	Industry	CD79	*Paenibacillus thermophilus*	Industry
CD28	*Clostridium aminovalericum*	BTM	CD80	*Proteus mirabilis*	BTM
CD29	*Clostridium cochlearium*	Industry	CD81, 82	*Proteus mirabilis*	BTM
CD30	*Clostridium magnum*	Industry	CD83	*B cereus HKG*	Industry
CD31	*C. pasteurianum/C. beijerinckii*	Industry	CD87-88	*Bacillus*	Industry
CD32	*C. pasteurianum/C. beijerinckii*	Industry	CD84-86 & CD89-CD101	*Bacillus licheniformis*	BTM
CD33	*Clostridium peptidivorans*	BTM	CD43	*Clostridium sporogenes*	Industry
CD34	*Clostridium peptidovorans DPC 7177*	BTM	CD46 & 47	*Clostridium tertium*	Industry
CD35	*Clostridium perfringens*	BTM	CD48	*Clostridium tetani*	BTM
CD36	*Clostridium perfringens*	BTM	CD49 &50	*Clostridium thiosulfatireducens DPC 7172*	Industry
CD37, 38, 40 & 41	*Clostridium saratogoforme*	BTM	CD51, 53-54 & 56-70 & 72-76	*Clostridium tyrobutyricum*	BTM
CD39	*Clostridium sartagoforme*	Industry	CD42, 44, 45 & 76	*Clostridium sporogenes*	BTM

*BTM, Bulk tank milk*.

Among the 101 isolates, the pathogens detected were *C. perfringens* and *C. tetani*. Although not a pathogen, the presence of *C. sporogenes* is notable in that it can be difficult to distinguish between *C. sporogenes* and *C. botulinum* because of the significant genomic synteny shared between the two species. *C. sporogenes* may also contribute to gas defects in continental style cheeses (Bermúdez et al., 2016). The presence of *C. tyrobutyricum, C. beijerinckii* and *C. tertium* is notable as these species have previously been associated with late blowing defects in cheese (Cocolin et al., 2004; Bermúdez et al., 2016). Other clostridia detected were *C. amygdalinum, C. bifermentans, C. algidcarnis, C. aminovelerium, C. peptidoveorans, C. sartagoforme, C. thiosulfatireducens, C. cochlearium*, and *C. celecrescens*. Of these, *C. bifermentans* has been associated with a pediatric infection previously (Brook, 1995) and both it and *C. cochlearium* have previously been isolated from powdered infant formula (Barash et al., 2010) as well as dairy farm effluent (Gupta and Brightwell, 2017), the latter observation potentially highlighting a source of these microbes in the dairy chain. To our knowledge, the presence of *C. amygdalinum, C. algidcarnis, C. aminovelerium, C. peptidoveorans, C. sartagoforme, C. thiosulfatireducens*, and *C. celecrescens* has not previously been reported in dairy sources.

Among the non-clostridia were 16 *Bacillus licheniformis* and 1 *B. cereus* strains. These are spoilage and pathogenic species, respectively, that have been associated with dairy foods (McHugh et al., 2017), bulk tank milk (BTM) (Miller et al., 2015; Sadiq et al., 2016) and dairy farm effluent (Gupta and Brightwell, 2017). Finally, three SRB isolates were identified as *P. mirabilis*. The detection of this Gram negative bacterium was unusual as it would be expected that *Proteus* would be inactivated by the heat-treatment step within the assay. Regardless, it is notable that *P. mirabilis* (Kawabata, 1980) and *B. licheniformis* (Harmon et al., 1971; Weenk et al., 1995; Fischer et al., 2012), though not *B. cereus*, have previously been found to cause false positive results in a SRC assay. Two *Paenibacillus spp*.(including *Paenibacillus thermophilus*) were isolated in this screen, this species has frequently been isolated from raw milk and processed dairy products previously (Ivy et al., 2012).

### SRB *In-silico* genome characterization

*In-silico* genome characterization was utilized to further investigate SRB taxonomy, and associated sulphite reducing genes. This analysis included genome sequences that were representative of the species detected in the dairy products and were already available on the NCBI database, as well as sequences corresponding to other cluster 1 *Clostridium*, including *C. botulinum* group and other known sulphite reducing *Clostridium spp*. and members of the *sensu stricto, Paenibacillus lactis* (due to the non-availability of a *P. thermophilus* genome sequence) and *Salmonella enterica typhimurium* LT2, as this species has both an *asrABC* gene cluster for dissimilatory sulphite reduction and a cys*IJ* operon for assimilatory sulphite reduction. Six additional *Clostridium* strains isolated from this study, i.e., *Clostridium aminovelericum* DPC 7173, *Clostridium thiosulfatireducens* DPC 7172, *Clostridium cochlerium* DPC 7174, *Clostridium tertium* DPC 7175, *Clostridium amygdalinum* DPC 7176 and *Clostridium peptidovorans* DPC 7177, were selected for whole genome sequencing due to the absence of existing whole genome sequences at the time of analysis. A list of all the genomes used for this analysis and a summary of the assembly statistics can be found in Table S1.

**Figure 1:**
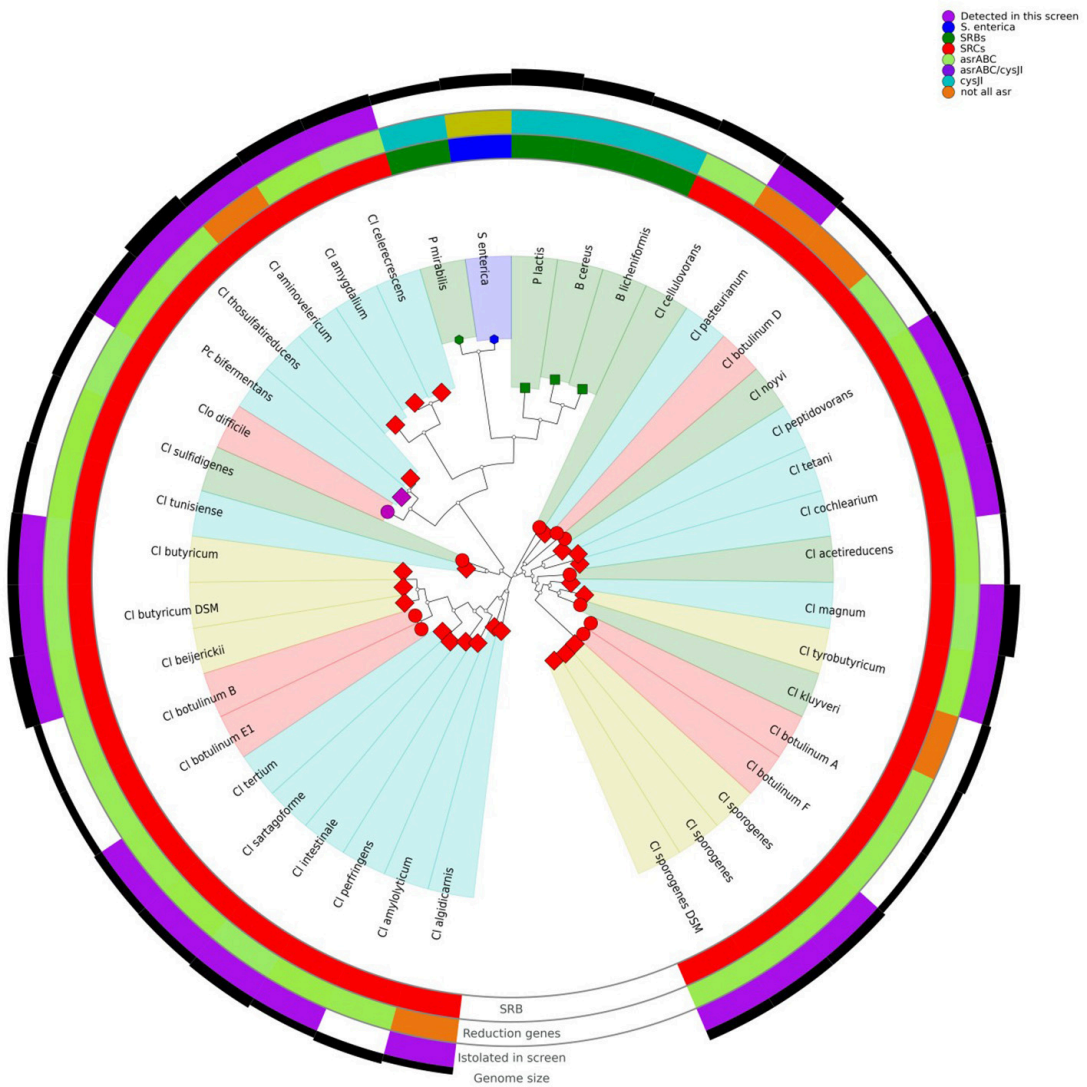
Phylophlan tree of SRBs, including those isolated during this screen, and presence/absence of specific sulphite reducing genes.

**Figure 2:**
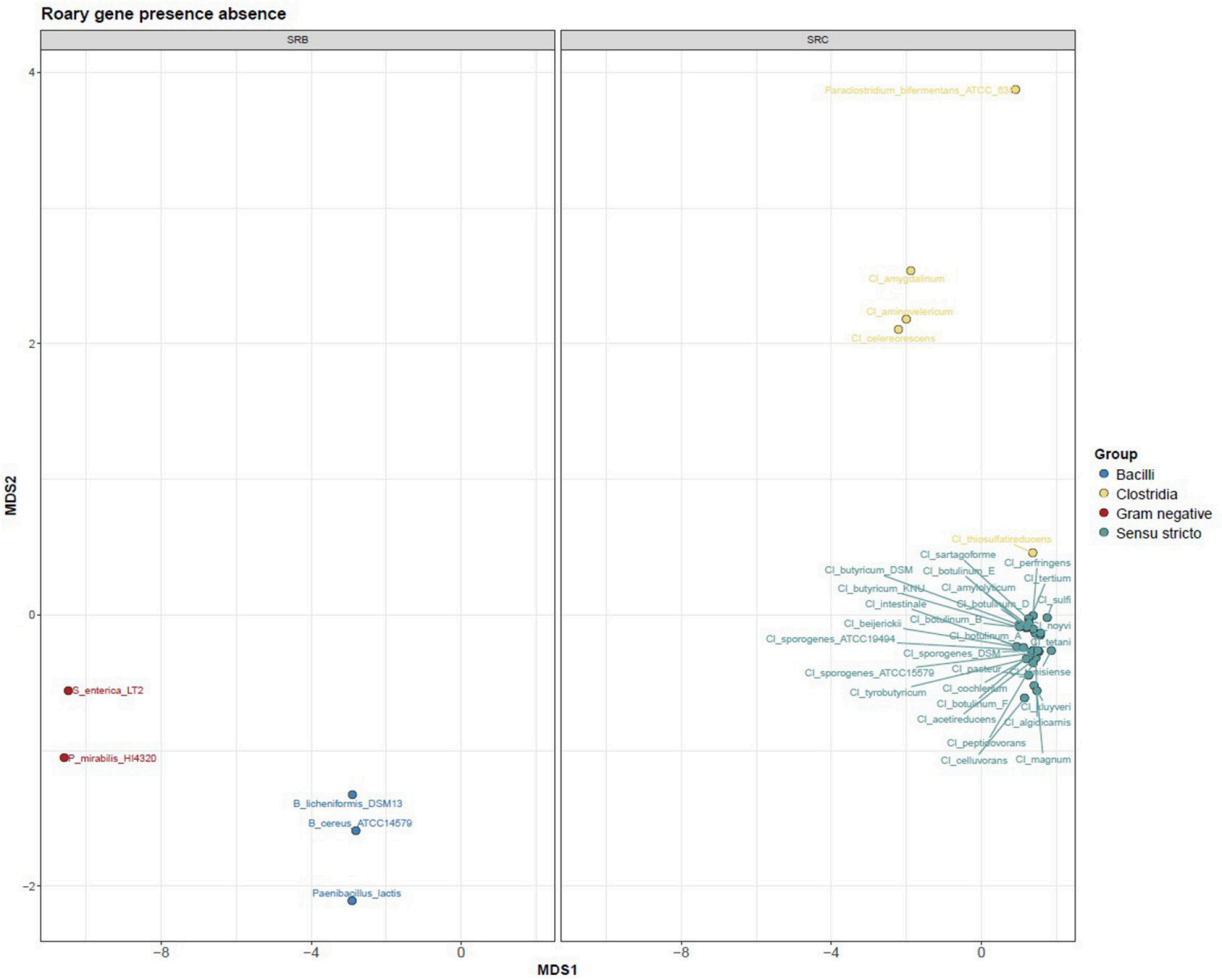
Bray Curtis PCoA profile depicting the dissimilarity of SRB genomes (faceted based on phylogeny).

After sequence assembly and annotation, global genome alignment was carried out using Phylophlan and Roary. Phylophlan uses 300 marker genes common to all bacteria, while Roary uses all the annotated genes of each genome. The Phylophlan tree (Figure 1) highlights the phylogenetic diversity which exists across bacteria with the SRB phenotype. The division between species that use the *cysJI* operon to reduce sulphite to sulfide (*Bacillus* spp., *P. lactis, P. mirabilis* and *S. enterica*) and those that use the *asr* operon is apparent. A similar functional separation, i.e., consistent with the presence or absence of *asrABC*, is observed in the gene presence absence Multidimensional scaling (MDS) plot generated from the Roary results (Figure 2). As noted, *S. enterica* has both *asrABC* and *cysJI* operons. In the Phylophlan tree, the newly sequenced *C. amygdalinum* and *C. aminovelericum* genomes cluster closely with that of *C. celecrescens*. In addition, *C. thiosulfatireducens* shows relatedness to *P. bifermentans* and *C. difficile*. These six species form a distinct branch which is distant from the rest of the *Clostridium* spp. Indeed, some of species falling within this subgroup have been recently reclassified, for instance the bacterium formally known as *Clostridium difficile* is now designated as *Clostridioides difficile* (Lawson et al., 2016). The separation of *C. amygdalinum* and *C. aminovelericum* from the *sensu stricto* can be seen in Figure 2. This is expected as they are members of *Clostridiu*m Clusters XIV and III respectively. It is also evident that the genomes of *C. tunisiense* and *C. sulfidigenes* form a distinct clade separate from the rest of the *sensu stricto Clostridium spp*. Neither of these bacteria were isolated during the present study but were included in the analysis as they are known to reduce sulphite (Thabet et al., 2004; Sallam and Steinbüchel, 2009). The distinct clustering of these strains was not anticipated and warrants future investigation. This separation of these two species in Figure 1 suggests that they belong to a distinct subcluster within the *sensu stricto*.

Both the Phylophlan tree and the MDS plot highlight the diversity of sulphite reducing microbes of interest to the dairy industry. While a great number of bacteria can reduce sulphite to sulphite via different pathways (Dahl et al., 2008), it would appear from these analyses that it is only bacteria which utilize the *asrABC* or the *cysIJ* operons which give a positive test for the SRC assay employed by dairy producers. More specifically, the clostridia that utilize the *asrABC* sulphite reduction pathway are of most concern as they include pathogenic and spoilage-associated bacteria belonging to the genus *Clostridium*. These results highlight the heterogeneity that exists within the Clostridia. While this has already been shown from the context of the 16S rRNA gene sequence (Wiegel et al., 2006), whole genome-wide heterogeneity has until now been examined for this Order of bacteria. Although many *Clostridium spp*. have been reclassified and placed with new or existing genera (Lawson et al., 2016), there is still an issue with *Clostridium* nomenclature. For instance, *C. aminovelericum* and *C. celecrescens* belong to Cluster III of the clostridia (Wiegel et al., 2006), while *C. amygdalinum* belongs to clostridia Cluster XIV based on its phylogeny and high GC content (Parshina et al., 2003). The SRC phenotype is distributed across this heterogeneous group of bacteria.

### Sulphite reducing protein homology in dairy-associated SRBs

**Figure 3:**
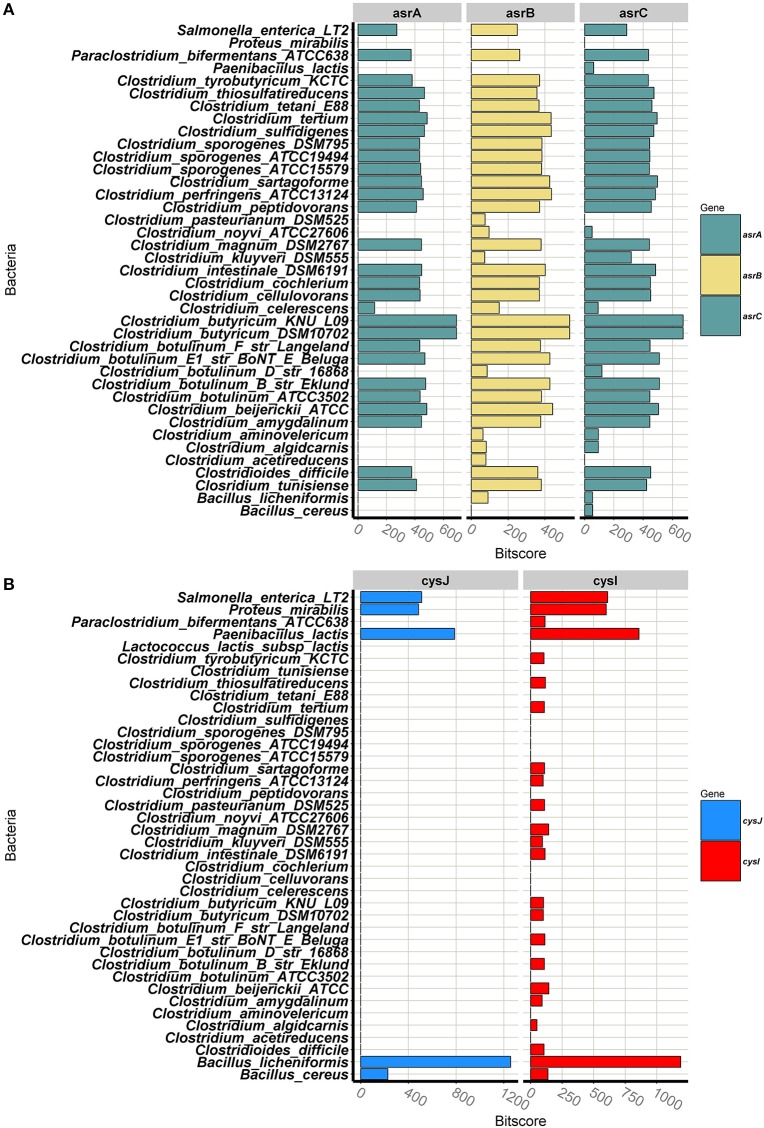
**(A)** Bar plot depicting bit-scores of BlastP query hits for *asrABC* from *C. butyricum*, and *fnt* from *C. perfringes*. **(B)** Bar plot depicting bit-scores of BlastP query hits for cysJI from *B. licheniformis*.

While the previous section examined the phylogeny of the SRC and SRB groups, this section details the homology associated with the proteins responsible for these phenotypes. Annotated complete sulphite reducing gene clusters for all the SRBs in the database can be seen in Figure S1B. Figure 3A depicts the BLASTp bit-score results for asrABC queries for all of the genomes in the constructed SRB database; the bit score is used to highlight proteins that are similar. The asrA protein sequence is present at a high degree of homology in the majority of *Clostridium* spp. and at a lower degree of homology in *C. celecresens*. Furthermore, there were no BLASTp hits for the asrA query for *C. acetireducens, C. algicarnis, C. aminovelericum, C. botulinum* D, *C. kluyveri, C. noyvi*, and *C. pasteurianum*. The asrB protein sequence was present in the all of the *Clostridium* genomes in the database (Figure S2). However, levels of homology found in *C. aetireducens, C. algicarnis, C. aminovelericum, C. botulinum* D, *C. kluyveri, C. noyvi*, and *C. pasteurianum* for this query were much lower than that within other *Clostridiu*m genomes. Similarly, for the asrC protein, the bit-scores for this query were again low for *C. algicarnis, C. aminovelericum, C. botulinum* D, *C. kluyveri*, and *C. noyvi* and no corresponding gene was found in the *C. acetireducens* and *C. pasteurianum* genomes. Furthermore, this sulphite reducing operon is present at a high degree of homology in *sensu stricto Clostridium* spp. Interestingly, *C. acetireducens, C. kluyveri, C. algicarnis, C. noyvi*, and *C. pasteurianum*, which are members of the *sensu stricto*, did not have the full operon based on these results and highlights that not all *asrABC* genes are necessary to confer this phenotype.

The bar plot in Figure 3B shows the BLASTp results for the cysJI queries for all genomes in the constructed SRB database. For the dissimilatory sulphite reducing pathway involving *cysJI*, the BLASTp bit-scores indicate that the cysJ gene is only present in *B. licheniformis, B. cereus, P. lactis, P. mirabilis*, and *S. enterica* and not in any of the other SRB genomes in the constructed database (Figure 3B). For the BLASTp with the cysI query, again the highest homology is shared with *B. licheniformis, P. lactis, P. mirabilis, S. enteria* and to a lesser extent *B. cereus*. The presence of this dissimilatory sulphite reductase gene cluster in these species is consistent with what is reported in the literature (Huang and Barrett, 1991).

The results from the BLASTp queries of the sulphite reducing genes of *Clostridium* prompted further examination of conserved amino acid domains with each amino acid sequence in this cluster. Conserved domains could act as targets for a nucleic acid-based detection assay for SRCs as an alternative to the non-specific agar-based approach. It was observed that asrA and C genes contain areas with conserved cysteine motifs. These 4Fe-4S clusters have been observed in asrA and C in *Salmonella* previously (Huang and Barrett, 1991). They have 4 conserved cysteine residues, with a proline toward the C terminus end of the domain. Amino acid sequence alignments can be seen in (Figure S3). The asrC protein also contains a siroheme binding site which is annotated in indigo (Figure S5). The asrB gene is involved in nucleotide binding (Ostrowski et al., 1989) (Figure S4). These alignments show the conservation in the functional regions of these genes. While similar functional domains might exist in other sulphite reducing bacteria using alternative pathways to the asrABC mediated reduction, the conserved proline appears to be a unique feature in the asr 4Fe-4S clusters. To verify that these conserved domains do not exist on other dairy associated SRBs, we examined the dissimilatory sulphite reducing genes in other SRBs from this analysis. The cysJI operon was also examined for conserved functional domains. The alignment for the alpha-subunit cysJ is shown in (Figure S6). The beta-subunit cysI contains a similar 4Fe-4S cluster to that in asrA and C (Figure S7). It is interesting to note that this sulphite binding cluster does not contain the conserved proline which is a feature of asr 4Fe-4S clusters. This shows that differences exist not only in the proteins used by these SRBs to reduce sulphite but also in the functional domains within this proteins compared to SRCs.

## Conclusion

Here, the extent to which the agar-based SRC assay fails to distinguish between SRCs and SRBs that are facultative anaerobes was the focus of an extensive investigation. It is apparent that there is a need for a more rapid assay with increased discriminatory power to distinguish between SRCs and the wider group of SRB. The failure of the conventionally applied culture based method to differentiate between Clostridia and other SRBs such as *Bacillus* and *Proteus* could potentially be overcome by applying a PCR based assay which targets the genetic basis for this phenotype in different microbes. Our genome-wide phylogenetic comparison of the dairy-associated SRB phenotype has shown the diversity that exists within this group of microbes. In addition to the noted distribution of this phenotype across Gram positive and negative bacteria, this phenotype is observed throughout the Order Clostridia, with isolates from the *sensu stricto, Lachnospiraceae* and Cluster XIV of the Clostridia all producing this phenotype. Furthermore, we have carried out a genomic characterization of the SRBs of interest to the dairy industry, with specific focus on Clostridia. This has highlighted the heterogeneity that exists within the SRC phenotype. The wider SRB phenotype can be divided into two further phenotypes based on each isolate's phylogeny and the pathway (*asrABC* or *cysJI*) they utilize to produce the sulphite reducing phenotype. While *asrABC* mediated sulphite reduction has been studied in *S. enterica* and *C. difficile*, it has not been previously examined in the context of SRC phenotype in the dairy industry. Here, we have carried out an *in-silico* screen for the genes of this operon in dairy-associated SRBs and have provided more clarity to what defines a SRC is on the basis of the presence or absence of the *asrABC* operon.

## Author contributions

PC and PO developed the concept with subsequent input from CD. CD carried out the research with input from PO and PC. CD drafted and edited the manuscript. Revised and edited by PO and PC.

### Conflict of interest statement

The authors declare that the research was conducted in the absence of any commercial or financial relationships that could be construed as a potential conflict of interest.

## References

[B1] Altschul S. F. Madden T. L. Schäffer A. A. Zhang J. Zhang Z. Miller W.. (1997). Gapped BLAST and PSI-BLAST: a new generation of protein database search programs. Nucleic Acids Res. 25, 3389–3402. 10.1093/nar/25.17.3389 9254694 PMC146917

[B2] Andrews S. (2010). FastQC: A Quality Control Tool for High Throughput Sequence Data. Available online at: https://www.bioinformatics.babraham.ac.uk/projects/fastqc/

[B3] Asnicar F. Weingart G. Tickle T. L. Huttenhower C. Segata N. (2015). Compact graphical representation of phylogenetic data and metadata with GraPhlAn. PeerJ 3:e1029. 10.7717/peerj.1029 26157614 PMC4476132

[B4] Barash J. R. Hsia J. K. Arnon S. S. (2010). Presence of soil-dwelling clostridia in commercial powdered infant formulas. J. Pediatr. 156, 402–408. 10.1016/j.jpeds.2009.09.072 20004414

[B5] Bassi D. Puglisi E. Cocconcelli P. S. (2015). Understanding the bacterial communities of hard cheese with blowing defect. Food Microbiol. 52, 106–118. 10.1016/j.fm.2015.07.004 26338123

[B6] Bennett S. D. Walsh K. A. Gould L. H. (2013). Foodborne disease outbreaks caused by *Bacillus cereus, Clostridium perfringens*, and *Staphylococcus aureus*—United States, 1998–2008. Clin. Infect. Dis. 57, 425–433. 10.1093/cid/cit244 23592829 PMC11334977

[B7] Bermúdez J. González M. J. Olivera J. A. Burgueño J. A. Juliano P. Fox E. M.. (2016). Seasonal occurrence and molecular diversity of clostridia species spores along cheesemaking streams of 5 commercial dairy plants. J. Dairy Sci. 99, 3358–3366. 10.3168/jds.2015-10079 26923043

[B8] Brook I. (1995). Clostridial infection in children. J. Med. Microbiol. 42, 78–82. 10.1099/00222615-42-2-78 7869351

[B9] Cocolin L. Innocente N. Biasutti M. Comi G. (2004). The late blowing in cheese: a new molecular approach based on PCR and DGGE to study the microbial ecology of the alteration process. Int. J. Food Microbiol. 90, 83–91. 10.1016/S0168-1605(03)00296-4 14672833

[B10] Czyzewski B. K. Wang D. N. (2012). Identification and characterization of a bacterial hydrosulphide ion channel. Nature 483, 494–497. 10.1038/nature10881 22407320 PMC3711795

[B11] Dahl C. Friedrich C. Kletzin A. (2008). Sulfur Oxidation in Prokaryotes. eLS.16924482

[B12] Doyle C. J. Gleeson D. Jordan K. Beresford T. P. Ross R. P. Fitzgerald G. F.. (2015). Anaerobic sporeformers and their significance with respect to milk and dairy products. Int. J. Food Microbiol. 197, 77–87. 10.1016/j.ijfoodmicro.2014.12.022 25574847

[B13] Doyle M. E. Glass K. (2013). Spores of Clostridium botulinum in Dried Dairy Products. Madison, WI: Food Research Institute.

[B14] Edgar R. C. (2004). MUSCLE: multiple sequence alignment with high accuracy and high throughput. Nucleic Acids Res. 32, 1792–1797. 10.1093/nar/gkh340 15034147 PMC390337

[B15] Fischer M. Zhu S. de Ree E. (2012). Culture media for the detection and enumeration of clostridia in food. Handb. Cult. Media Food Water Microbiol. 66–89. 10.1039/9781847551450-00066

[B16] Guillouard I. Auger S. Hullo M. F. Chetouani F. Danchin A. Martin-Verstraete I. (2002). Identification of *Bacillus subtilis* CysL, a regulator of the cysJI operon, which encodes sulfite reductase. J. Bacteriol. 184, 4681–4689. 10.1128/JB.184.17.4681-4689.2002 12169591 PMC135269

[B17] Gupta T. B. Brightwell G. (2017). Farm level survey of spore-forming bacteria on four dairy farms in the Waikato region of New Zealand. Microbiologyopen 6:e00457. 10.1002/mbo3.457 28256808 PMC5552919

[B18] Harmon S. M. Kautter D. A. Peeler J. T. (1971). Improved medium for enumeration of *Clostridium perfringens*. Appl. Microbiol. 22, 688–692. 4331774 10.1128/am.22.4.688-692.1971 PMC376387

[B19] Huang C. J. Barrett E. L. (1991). Sequence analysis and expression of the *Salmonella typhimurium* asr operon encoding production of hydrogen sulfide from sulfite. J. Bacteriol. 173, 1544–1553. 10.1128/jb.173.4.1544-1553.1991 1704886 PMC207294

[B20] ICMSF (2014). Usefulness of Testing for Clostridium botulinum in Powdered Infant Formula and Dairy-based Ingredients for Infant Formula. ICMSF.

[B21] Ivy R. A. Ranieri M. L. Martin N. H. den Bakker H. C. Xavier B. M. Wiedmann M.. (2012). Identification and characterization of psychrotolerant sporeformers associated with fluid milk production and processing. Appl. Environ. Microbiol. 78, 1853–1864. 10.1128/AEM.06536-11 22247129 PMC3298126

[B22] Kawabata N. (1980). Studies on the sulfite reduction test for clostridia. Microbiol. Immunol. 24, 271–279. 10.1111/j.1348-0421.1980.tb02830.x 6993870

[B23] Kelley L. A. Mezulis S. Yates C. M. Wass M. N. Sternberg M. J. (2015). The Phyre2 web portal for protein modeling, prediction and analysis. Nat. Protoc. 10, 845–858. 10.1038/nprot.2015.053 25950237 PMC5298202

[B24] Lawson P. A. Citron D. M. Tyrrell K. L. Finegold S. M. (2016). Reclassification of Clostridium difficile as *Clostridioides difficile* (Hall and O'Toole 1935) Prévot 1938. Anaerobe 40, 95–99. 10.1016/j.anaerobe.2016.06.008 27370902

[B25] Li H. Handsaker B. Wysoker A. Fennell T. Ruan J. Homer N.. (2009). The sequence alignment/map format and SAMtools. Bioinformatics 25, 2078–2079. 10.1093/bioinformatics/btp352 19505943 PMC2723002

[B26] Ludwig W. Schleifer K. H. Whitman W. B. (2009). Revised road map to the phylumFirmicutes, in Bergey's Manual® of Systematic Bacteriology, eds Vos P. Garrity G. Jones D. Krieg N. R. Ludwig W. Rainey F. A. Schleifer K.-H. Whitman W. (New York, NY: Springer), 1–13.

[B27] McAuley C. M. McMillan K. Moore S. C. Fegan N. Fox E. M. (2014). Prevalence and characterization of foodborne pathogens from Australian dairy farm environments. J. Dairy Sci. 97, 402–412. 10.3168/jds.2014-8735 25282417

[B28] McHugh A. J. Feehily C. Hill C. Cotter P. D. (2017). Detection and enumeration of spore-forming bacteria in powdered dairy products. Front. Microbiol. 8:109. 10.3389/fmicb.2017.00109 28197144 PMC5281614

[B29] Miller R. A. Kent D. J. Watterson M. J. Boor K. J. Martin N. H. Wiedmann M. (2015). Spore populations among bulk tank raw milk and dairy powders are significantly different. J. Dairy Sci. 98, 8492–8504. 10.3168/jds.2015-9943 26476952

[B30] Ostrowski J. Barber M. J. Rueger D. E. Miller B. E. Siegel L. M. Kredich N. M. (1989). Characterization of the flavoprotein moieties of NADPH-sulfite reductase from *Salmonella typhimurium* and *Escherichia coli*. Physicochemical and catalytic properties, amino acid sequence deduced from DNA sequence of cysJ, and comparison with NADPH-cytochrome P-450 reductase. J. Biol. Chem. 264, 15796–15808. 2550423

[B31] Page A. J. Cummins C. A. Hunt M. Wong V. K. Reuter S. Holden M. T.. (2015). Roary: rapid large-scale prokaryote pan genome analysis. Bioinformatics 31, 3691–3693. 10.1093/bioinformatics/btv421 26198102 PMC4817141

[B32] Parshina S. N. Kleerebezem R. Sanz J. L. Lettinga G. Nozhevnikova A. N. Kostrikina N. A. (2003). *Soehngenia saccharolytica* gen. nov., sp. nov. and *Clostridium amygdalinum* DPC 7176 sp. nov., two novel anaerobic, benzaldehyde-converting bacteria. Int. J. Syst. Evol. Microbiol. 53, 1791–1799. 10.1099/ijs.0.02668-0 14657106

[B33] Peng Y. Leung H. C. Yiu S. M. Chin F. Y. (2012). IDBA-UD: a de novo assembler for single-cell and metagenomic sequencing data with highly uneven depth. Bioinformatics 28, 1420–1428. 10.1093/bioinformatics/bts174 22495754

[B34] Price M. N. Dehal P. S. Arkin A. P. (2009). FastTree: computing large minimum evolution trees with profiles instead of a distance matrix. Mol. Biol. Evol. 26, 1641–1650. 10.1093/molbev/msp077 19377059 PMC2693737

[B35] Sadiq F. A. Li Y. Liu T. Flint S. Zhang G. Yuan L.. (2016). The heat resistance and spoilage potential of aerobic mesophilic and thermophilic spore forming bacteria isolated from Chinese milk powders. Int. J. Food Microbiol. 238, 193–201. 10.1016/j.ijfoodmicro.2016.09.009 27657656

[B36] Sallam A. Steinbüchel A. (2009). *Clostridium sulfidigenes* sp. nov., a mesophilic, proteolytic, thiosulfate-and sulfur-reducing bacterium isolated from pond sediment. Int. J. Syst. Evol. Microbiol. 59, 1661–1665. 10.1099/ijs.0.004986-0 19542123

[B37] Scallan E. Hoekstra R. M. Angulo F. J. Tauxe R. V. Widdowson M. A. Roy S. L.. (2011). Foodborne illness acquired in the United States—major pathogens. Emerg. Infect. Dis. 17, 7–15. 10.3201/eid1701.P11101 21192848 PMC3375761

[B38] Seemann T. (2014). Prokka: rapid prokaryotic genome annotation. Bioinformatics 30, 2068–2069. 10.1093/bioinformatics/btu153 24642063

[B39] Segata N. Börnigen D. Morgan X. C. Huttenhower C. (2013). PhyloPhlAn is a new method for improved phylogenetic and taxonomic placement of microbes. Nat. Commun. 4:2304. 10.1038/ncomms3304 23942190 PMC3760377

[B40] Simpson P. J. Stanton C. Fitzgerald G. F. Ross R. P. (2003). Genomic diversity and relatedness of bifidobacteria isolated from a porcine cecum. J. Bacteriol. 185, 2571–2581. 10.1128/JB.185.8.2571-2581.2003 12670982 PMC152629

[B41] Standards I. O. O. (2003). Microbiology of Food and Animal Feeding Stuffs - Horizontal Method for the Enumeration of Sulfite-Reducing Bacteria Growing Under Anaerobic Conditions. ISO 2003.

[B42] Standards I. O. O. (2004). Microbiology of Food And Animal Feeding Stuffs – Horizontal Method for the Enumeration of Clostridium perfringens – Colony-count Technique. ISO 2004.

[B43] Sugiyama H. (1951). Studies on factors affecting the heat resistance of spores of *Clostridium botulinum*. J. Bacteriol. 62, 81–96. 14861162 10.1128/jb.62.1.81-96.1951 PMC386087

[B44] Thabet O. B. D. Fardeau M.-L. Joulian C. Thomas P. Hamdi M. Garcia J.-L.. (2004). Clostridium tunisiense sp. nov., a new proteolytic, sulfur-reducing bacterium isolated from an olive mill wastewater contaminated by phosphogypse. Anaerobe 10, 185–190. 10.1016/j.anaerobe.2004.04.002 16701517

[B45] Turnbull A. L. Surette M. G. (2008). L-Cysteine is required for induced antibiotic resistance in actively swarming *Salmonella enterica* serovar typhimurium. Microbiology 154, 3410–3419. 10.1099/mic.0.2008/020347-0 18957594

[B46] Waterhouse A. M. Procter J. B. Martin D. M. Clamp M. Barton G. J. (2009). Jalview Version 2—a multiple sequence alignment editor and analysis workbench. Bioinformatics 25, 1189–1191. 10.1093/bioinformatics/btp033 19151095 PMC2672624

[B47] Weenk G. Fitzmaurice E. Mossel D. (1991). Selective enumeration of spores of *Clostridium* species in dried foods. J. Appl. Microbiol. 70, 135–143. 2019549 10.1111/j.1365-2672.1991.tb04439.x

[B48] Weenk G. Van den Brink J. Struijk C. Mossel D. (1995). Modified methods for the enumeration of spores of mesophilic Clostridium species in dried foods. Int. J. Food Microbiol. 27, 185–200. 10.1016/0168-1605(94)00164-2 8579989

[B49] Wells-Bennik M. H. Driehuis F. van Hijum S. A. (2016). Prospects for improved control of dairy-relevant sporeformers using-omics technologies. Curr. Opin. Food Sci. 10, 38–44. 10.1016/j.cofs.2016.08.005

[B50] Wiegel J. Tanner R. Rainey F. A. (2006). An introduction to the family *Clostridiaceae”* in The Prokaryotes, eds Dworkin M. Falkow S. Rosenberg E. Schleifer K. H. Stackebrandt E. (New York, NY: Springer), 654–678.

