# Supplementary material for: Genomic Characterization of Sulphite Reducing Bacteria Isolated From the Dairy Production Chain
Source: Front Microbiol. 2018 Jul 5;9:1507. doi: 10.3389/fmicb.2018.01507 (PMC6041559; doi:10.3389/fmicb.2018.01507)
Supplement: Supplementary file 1 [file Image_1.PDF]

Table S1: Annotated genomes utilised to facilitate an *in silico* screen for sulphite reducing genes

**Species**	**Strain**	**GC %**	**Size Mb**
Bacillus cereus	ATCC 14579	35.3	5.42
Bacillus licheniformis	DSM 13	46.2	4.22
Clostridium acetireducens	DSM 10703	26.7	2.4
Clostridium algidcarnis	B3	3.06	30.3
Clostridium beijerickii	ATCC 35702	30	6.49
Clostridium botulinum	B str Eklund	27.47	3.8
Clostridium botulinum	ATCC 3502	28.19	3.9
Clostridium botulinum	D str 16868	28.17	3.08
Clostridium botulinum	E1 str BoNT E Beluga	27	3.99
Clostridium botulinum	F str Langeland	28.29	4.01
Clostridium butyricum	DSM 10702	28.5	4.59
Clostridium butyricum	KNU L09	32	3.82
Clostridium celerescens	AAU1	27.9	3.98
Clostridium celluvorans	743B	31.2	5.26
Clostridium intestinale	DSM 6191	30.1	4.6
Clostridium kluyveri	DSM 555	32.02	4.02
Clostridium magnum	DSM 2767	32.1	6.63
Clostridium noyvi	ATCC 27606	27.57	2.61
Clostridium pasteurianum	DSM 525	29.9	4.35
Clostridium perfringens	ATCC 13124	28.4	3.26
Clostridium sartagoforme	AAU1	27.9	3.98
Clostridium sporogenes	ATCC 15579	28	4.1
Clostridium sporogenes	ATCC 19494	27.9	4.06
Clostridium sporogenes	DSM 795	28	4.14
Clostridium sulfidigenes	113A c1	30	3.72
Clostridium tetani	E88	28.8	2.87
Clostridium tunisiense	TJ C661	31.2	4.31
Clostridium tyrobutyricum	KCTC	31.1	3.13
Clostridioides difficile	630	29.1	4.2
Paenibaciilus lactis	154	51.8	6.8
Proteus mirabilis	HI4320	38.8	4.09
Paraclostridium bifermentans	ATCC 638	28.4	3.6
Clostridium amygdalinum	DPC 7176	40	5.3
Clostridium peptidovorans	DPC 7177	33	3.4
Clostridium aminovelericum	DPC 7173	35	4.4
Clostridium thiosulfatireducens	DPC 7172	28.3	6.7
Clostridium cochlearium	DPC 7174	29.2	2.6
Clostridium tertium	DPC 7175	28.9	3.6
Salmonella enterica LT2	LT2	52.2	4.95

Table S1: Roary core genes of SRC phenotype

Gene	Annotation	No. isolates
accB	Biotin carboxyl carrier protein of acetyl-CoA carboxylase	33
lon1	Lon protease 1	33
clpC	Negative regulator of genetic competence ClpC/MecB	33
mraY	Phospho-N-acetylmuramoyl-pentapeptide-transferase	33
rpe	Ribulose-phosphate 3-epimerase	33
clpP	ATP-dependent Clp protease proteolytic subunit	33
fmt	Methionyl-tRNA formyltransferase	33
pnp	Polyribonucleotide nucleotidyltransferase	33
trmD	tRNA (guanine-N(1)-)-methyltransferase	33
murB	UDP-N-acetylenolpyruvoylglucosamine reductase	33
group_2420	Radical SAM superfamily protein	33
prkC	Serine/threonine-protein kinase PrkC	33
mazG	Nucleoside triphosphate pyrophosphohydrolase	33
fabD	Malonyl CoA-acyl carrier protein transacylase	33
fabG_2	3-oxoacyl-[acyl-carrier-protein] reductase FabG	33
fabF	3-oxoacyl-[acyl-carrier-protein] synthase 2	33
accC	Biotin carboxylase	33
uppS	Ditrans,polycis-undecaprenyl-diphosphate synthase ((2E,6E)-farnesyl-diphosphate specific)	33
ispG	4-hydroxy-3-methylbut-2-en-1-yl diphosphate synthase	33
uvrC	UvrABC system protein C	33
clpX	ATP-dependent Clp protease ATP-binding subunit ClpX	33
prfB	Peptide chain release factor 2	33
rlmN	putative dual-specificity RNA methyltransferase RlmN	33
miaA	tRNA dimethylallyltransferase	33
pgsA	CDP-diacylglycerol--glycerol-3-phosphate 3-phosphatidyltransferase	33
rimO	Ribosomal protein S12 methylthiotransferase RimO	33
group_2719	dihydrolipoamide dehydrogenase	33
dxr	1-deoxy-D-xylulose 5-phosphate reductoisomerase	33
group_2821	glmZ(sRNA)-inactivating NTPase	33
ispH	hypothetical protein	33
dxs_1	1-deoxy-D-xylulose-5-phosphate synthase	33
group_2882	hypothetical protein	33
yrrK	Putative Holliday junction resolvase	33
dnaK_1	Chaperone protein DnaK	33
rpsT	30S ribosomal protein S20	33
gpmI	2,3-bisphosphoglycerate-independent phosphoglycerate mutase	33
topA	DNA topoisomerase 1	33
mnmG	tRNA uridine 5-carboxymethylaminomethyl modification enzyme MnmG	33
rsmG	Ribosomal RNA small subunit methyltransferase G	33

rsmH	Ribosomal RNA small subunit methyltransferase H	33
engB	putative GTP-binding protein EngB	33
group_2983	Nucleoid-associated protein	33
yvyD	Putative sigma-54 modulation protein	33
tepA	Translocation-enhancing protein TepA	33
mdeA	methionine gamma-lyase	33
group_3095	alanine racemase	33
rpsR	30S ribosomal protein S18	33
hpt_2	Hypoxanthine-guanine phosphoribosyltransferase	33
tpiA	Triosephosphate isomerase	33
rex	Redox-sensing transcriptional repressor Rex	33
frr	Ribosome-recycling factor	33
group_3184	R3H domain protein	33
tig	Trigger factor	33
recR	Recombination protein RecR	33
prfA	Peptide chain release factor 1	33
greA_1	Transcription elongation factor GreA	33
rpsP	30S ribosomal protein S16	33
sigF_2	RNA polymerase sigma-F factor	33
ssbB	Single-stranded DNA-binding protein SsbB	33
group_3473	hypothetical protein	33
tsf	Elongation factor Ts	33
dnaA	Chromosomal replication initiator protein DnaA	33
sigE	RNA polymerase sigma-E factor precursor	33
pyrB	Aspartate carbamoyltransferase catalytic chain	33
rpoB	DNA-directed RNA polymerase subunit beta	33
group_3678	hypothetical protein	33
rpsO	30S ribosomal protein S15	33
ffh	Signal recognition particle protein	33
lepA	Elongation factor 4	33
fabZ	3-hydroxyacyl-[acyl-carrier-protein] dehydratase FabZ	33
rpmF	50S ribosomal protein L32	33
sigF_1	RNA polymerase sigma-F factor	33
rplK	50S ribosomal protein L11	33
group_4134	hypothetical protein	33
glyQS	Glycine--tRNA ligase	33
group_4434	hypothetical protein	33
group_4475	hypothetical protein	33
rpmB	50S ribosomal protein L28	33
def	Peptide deformylase	33
ftsH_2	ATP-dependent zinc metalloprotease FtsH	33
accD	Acetyl-coenzyme A carboxylase carboxyl transferase subunit beta	33

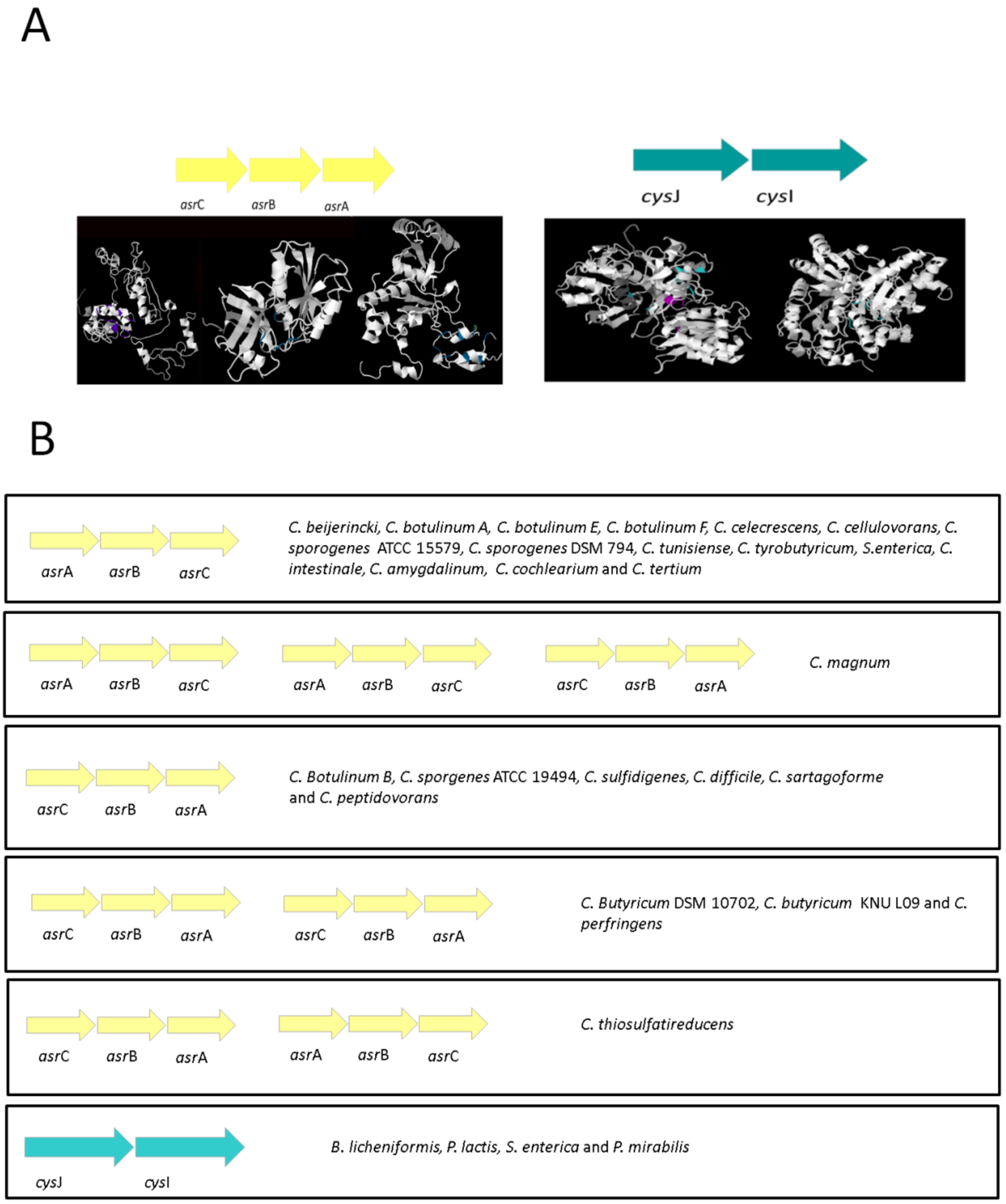

Fig.S1: (A) *asrABC* and *cysJI* sulphite reducing operons, with tertiary structures of proteins, (B) annotated complete sulphite reducing gene clusters in SRBs

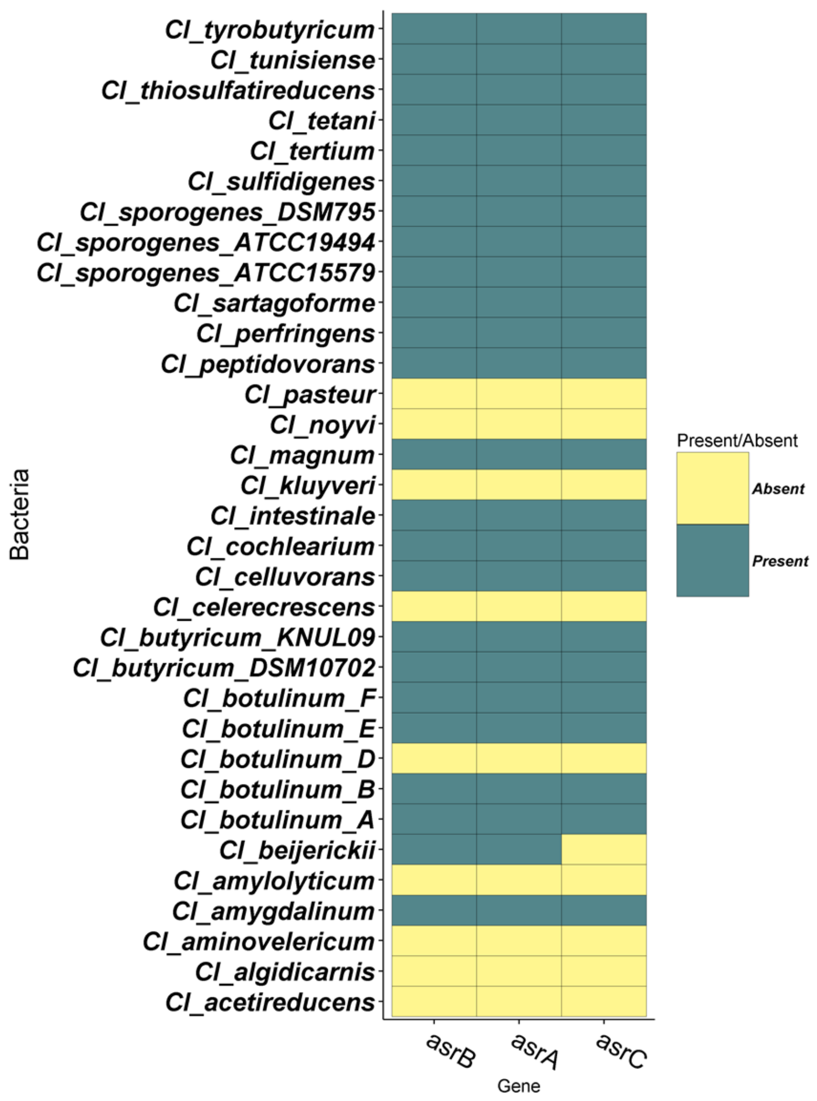

Fig.S2: Roary results for sulphite reducing genes

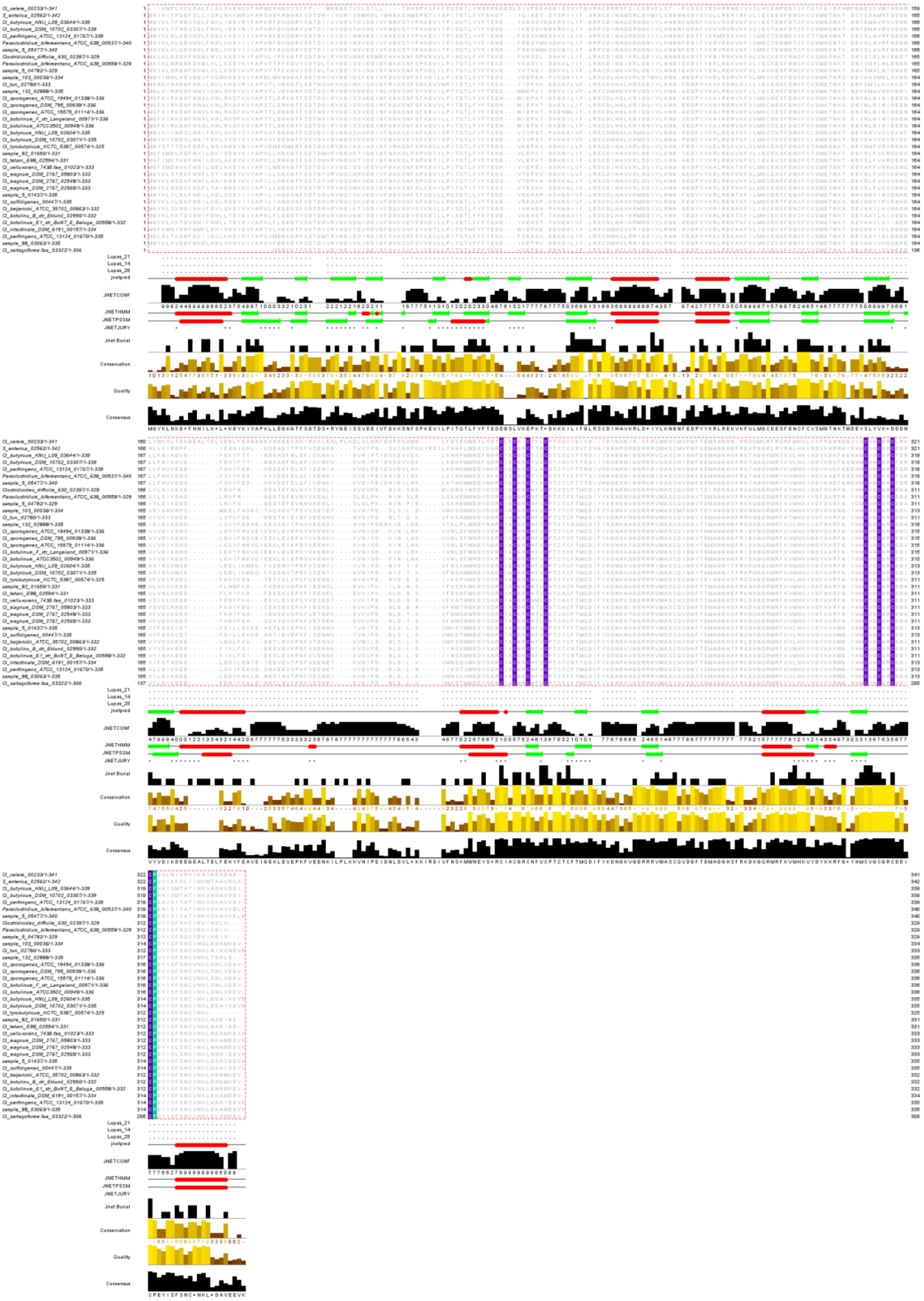

Fig.S3: Jalview alignment of *asrA* gene based on blastp results; the conserved cysteine and proline residues are highlighted

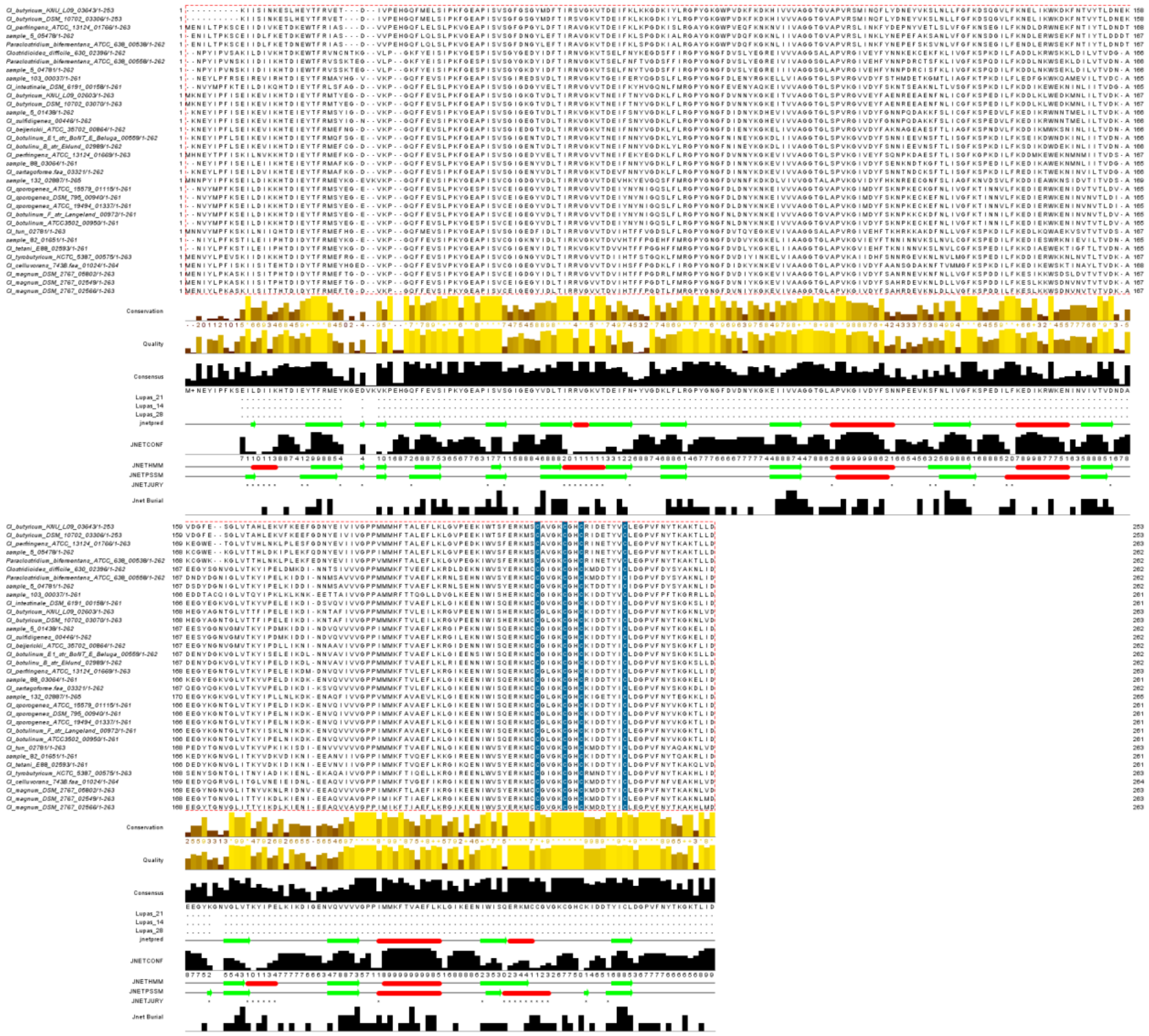

Fig.S4: Jalview alignment of *asrB* gene based on blastp results; conserved cysteine residues from this domain are highlighted in blue in the jalview alignments

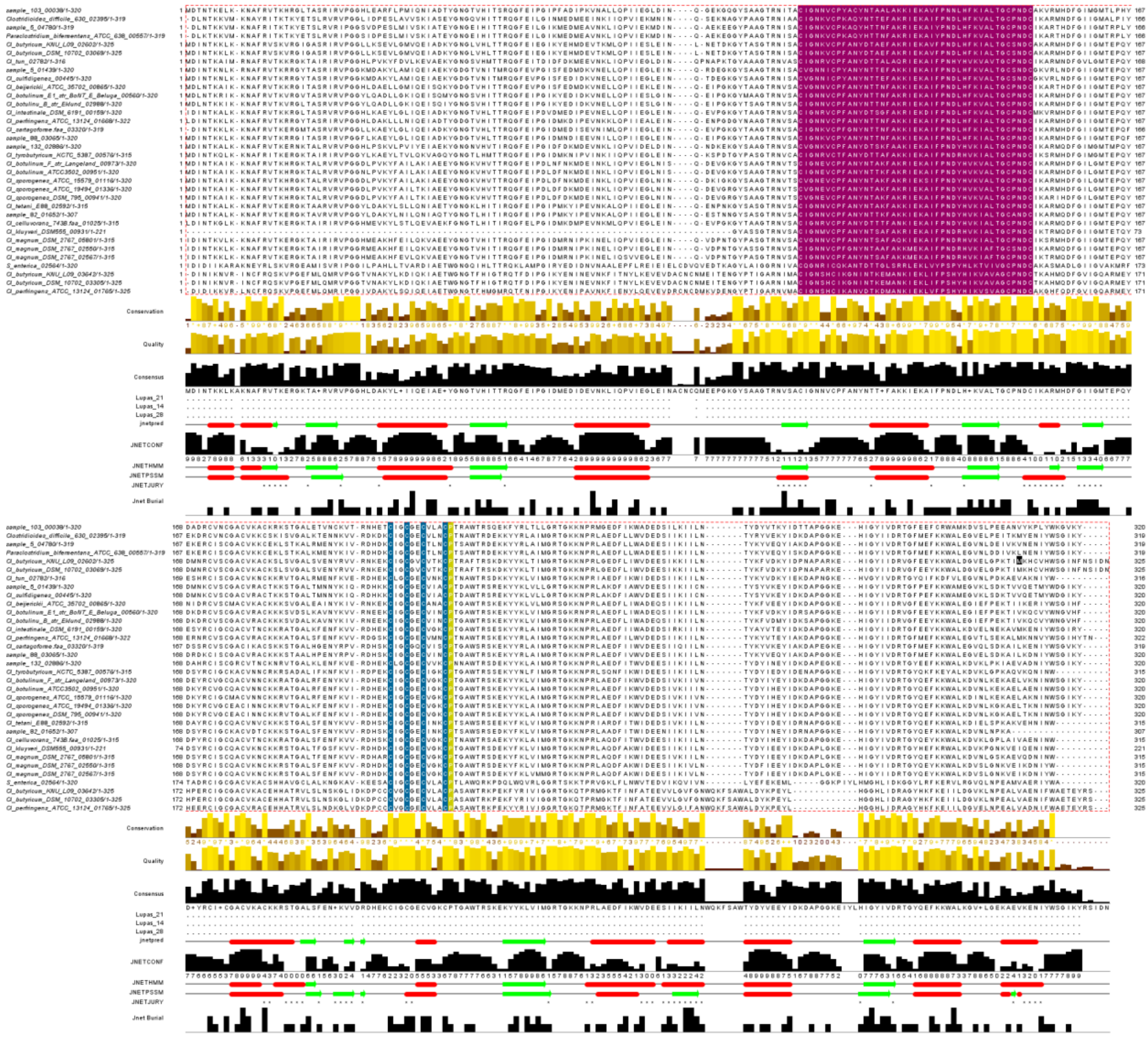

Fig.S5: Jalview alignment of *asrC* gene based on blastp results; the conserved siroheme binding site and conserved cysteine and proline residues are highlighted

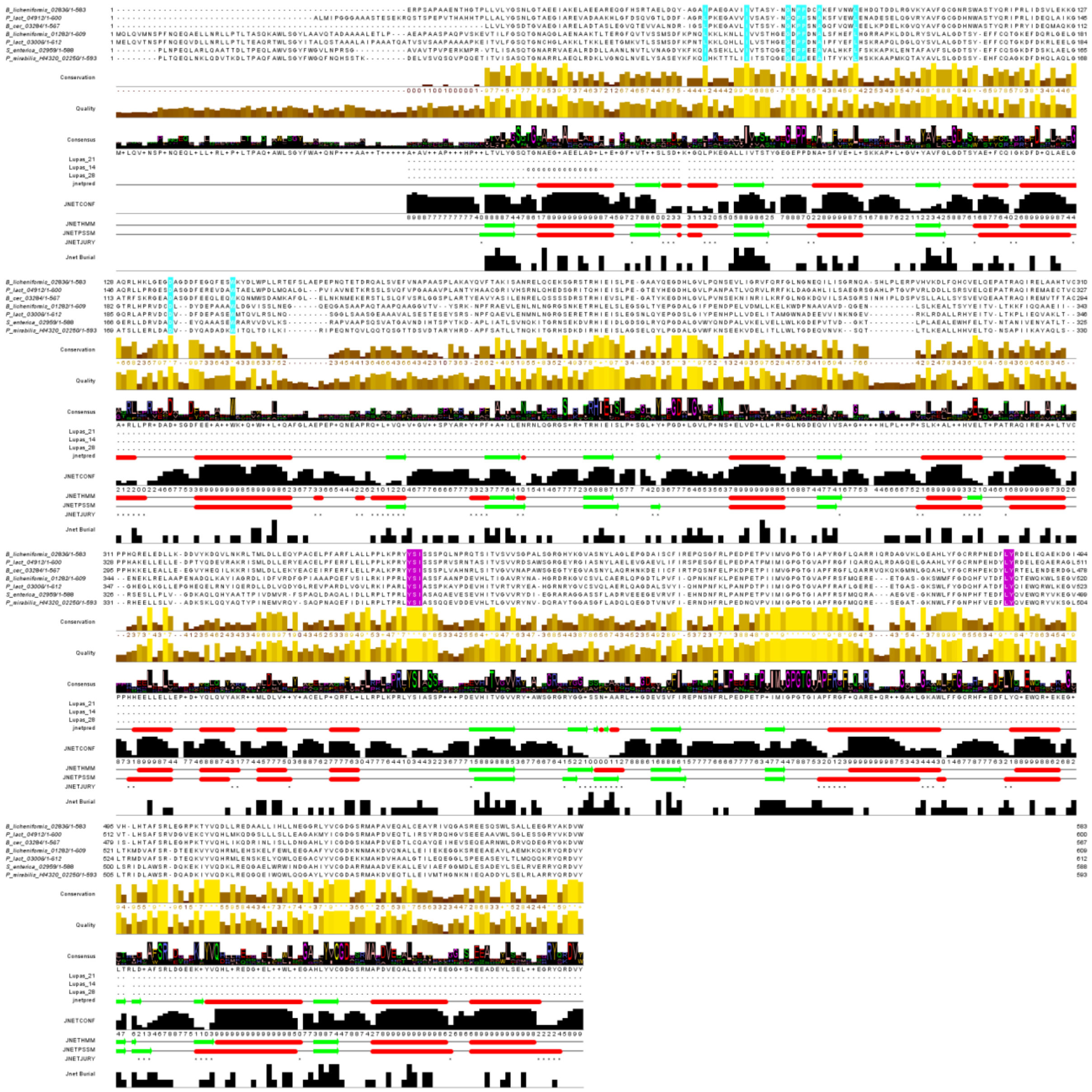

Fig.S6: Jalview alignment of *cysJ* gene based on blastp results; conserved amino acids in the mononucleotide binding domains, highlighted in the light blue

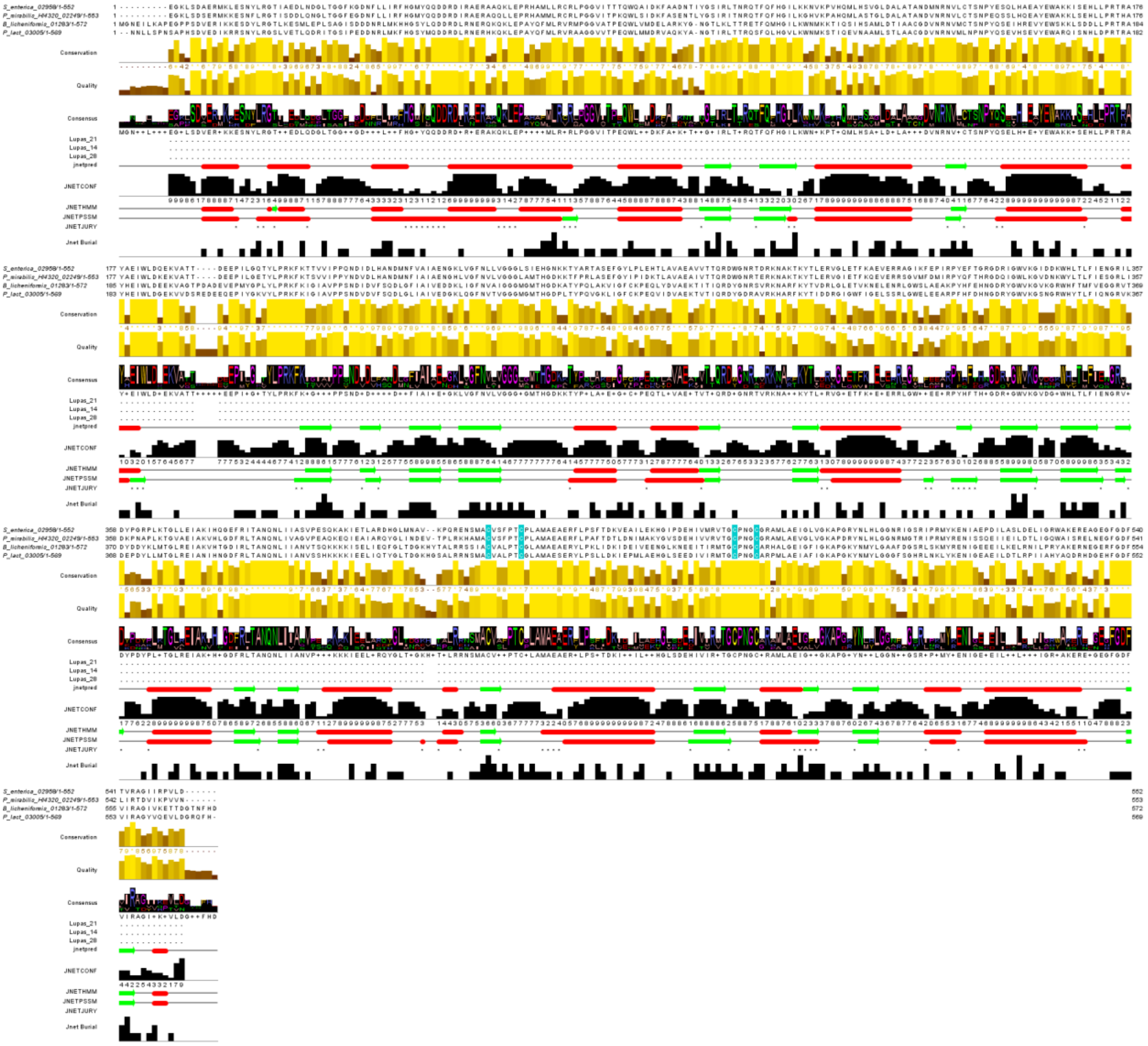

Fig.S7: Jalview alignment of *cysI* gene based on blastp results; conserved cysteine residues in this amino acid sequence are highlighted
